# Reliability and validity of the Sinhala version of the physical activity questionnaire for older children (PAQ-C)

**DOI:** 10.1371/journal.pone.0343709

**Published:** 2026-03-23

**Authors:** E. Liyanage, C. Jayasinghe, I. Liyanage

**Affiliations:** 1 Department of Physiotherapy, Faculty of Allied Health Sciences, University of Peradeniya, Peradeniya, Sri Lanka; 2 Department of Physical Therapy, Niigata University of Health and Welfare, Niigata, Japan; UBC: The University of British Columbia, CANADA

## Abstract

**Introduction:**

In Sri Lanka, as non-communicable diseases rise, the significance of physical activity is increasingly recognised, particularly in children. The Physical Activity Questionnaire for Older Children (PAQ-C) is widely used and can be considered as one of the best available tools for assessing physical activity in children. However, a validated tool for Sinhala-speaking children in Sri Lanka has been lacking. This study aims to culturally adapt and validate the PAQ-C for Sinhala-speaking children and evaluate its psychometric properties.

**Methodology:**

This cross-sectional study involved 301 schoolchildren (197 males and 104 females) aged 8–11 years, during which the PAQ-C was translated into Sinhala, and a pre-final version was developed. In terms of validity, content validity, that is, the degree to which the content of an instrument is an adequate reflection of the construct to be measured, was assessed using the content validity index. In terms of reliability, test-retest reliability was measured with the intraclass correlation coefficient (ICC).

**Results:**

The Sinhala version of the PAQ-C demonstrated CVI at both item and scale levels is as one. The ICC was acceptable at 0.83.

**Conclusion:**

The Sinhala version of the PAQ-C demonstrates good content validity and acceptable test–retest reliability, affirming its suitability as a cost-effective tool for assessing physical activity levels among Sinhala-speaking children in Sri Lanka and supporting informed interventions and public health strategies in the country.

## Introduction

The increasing prevalence of digital technologies and screen-based entertainment has contributed to a noticeable decline in physical activity among children [1, 2]. Furthermore, the growing preference for transportation by car and other motor vehicles has reduced active modes of transportation such as walking and cycling, further diminishing physical activity levels [3]. A similar pattern is observed in Sri Lanka, particularly in urban areas like Colombo [4]. Although data at the national level for children aged 8–12 remain limited, regional studies consistently highlight insufficient physical activity among Sri Lankan schoolchildren [5, 6]. Both the Rathnapura and Kalutara studies applied the WHO-recommended threshold of at least 60 minutes of moderate-to-vigorous physical activity per day for children aged 5–17 [7]. In Rathnapura District, 72.5% of 13–14-year-olds did not meet this benchmark, with girls and urban children showing particularly low engagement [8]. Similarly, in Kalutara District, 76.4% of 14–15-year-olds fell short of the same threshold, indicating widespread sedentary behaviour [9].

Low physical activity levels in children are associated with increased risks of obesity, cardiovascular disease, and metabolic conditions like type 2 diabetes [10]. Children who engage in insufficient physical activity may be at increased risk of developing chronic health conditions later in life [11]. Regular physical activity helps children to improve their cardiovascular and muscular fitness and also helps to manage body weight. Other than these physical well-being, it also helps to improve psychological well-being and reduce the risk of developing chronic diseases such as diabetes mellitus type II, hypertension, cancer-related diseases, and lower peak bone mass [12].

Valid and reliable assessment tools are required to gain insight into the level of physical activity and the health benefits and issues related to it. A variety of instruments exist for evaluating physical activity. Objective measurement of physical activity is often carried out using devices like accelerometers and pedometers [13]. However, these tools are very expensive and also not feasible for use among large populations. Moreover, objective tools often face compliance challenges; for instance, children may forget to wear them consistently [14]. Additionally, objective tools may also underestimate certain types of physical activity, such as cycling and resistance training, due to limited detection of non-ambulatory movements [15, 16]. There are self-reporting measures used to estimate the physical activity level, such as self-report questionnaires and self-report activity diaries/ logs [13]. Self-administered questionnaires offer a practical and cost-effective method for assessing physical activity, particularly in large populations, as they require minimal logistical support and participant burden [17]. Additionally, such questionnaires can be completed in a brief timeframe. Thus, those tools make it possible to investigate a large sample of children within a short time period [18].

The Physical Activity Questionnaire (PAQ) series comprises validated self-report instruments for assessing general physical activity levels in youth populations. Specifically, the PAQ-A (Physical Activity Questionnaire for Adolescents) targets adolescents aged 14–20 years [19], while the PAQ-C (Physical Activity Questionnaire for Older Children) is designed for older children aged approximately 8–14 years [20]. PAQ-C is a self-administered, 7-day recall questionnaire which can be used to evaluate school and leisure time physical activity levels [21]. The PAQ-C comprises ten items: nine core items rated on a five-point Likert scale, designed to capture overall physical activity levels and a final question that determines whether the reported week was typical or influenced by illness or other factors affecting usual activity level. Its format allows for easy administration within classroom settings, making it suitable for school-based assessments [22]. The questionnaire captures children’s activity behaviours across multiple timeframes spanning school hours, after-school periods, and weekends to provide a comprehensive view of weekly physical activity patterns. In general, the PAQs have shown relatively high correlation coefficients with alternative physical activity measures compared to other recall measures [21]. The Physical Activity Questionnaire for Older Children (PAQ-C) is widely used and can be considered as one of the best available tools for assessing physical activity in children [23]. Evidence shows that PAQ-C has been translated into numerous languages, such as Japanese [14], Chinese [24], Malay [25], Dutch [19], Spanish [26], and Turkish [27], and the pooled construct validity of the PAQ-C varies across its cross-culturally adapted language versions, ranging from very low to high [23]. The PAQ-C also shows acceptable test–retest reliability (ICC ranging from 0.70–0.82), with results depending on retest interval, language version, and study quality [28]. Furthermore, PAQ-C can be used to assess the physical activity in obese and overweight children [29].

There is a lack of reliable and valid assessment tools to evaluate the physical activity level among school children in Sri Lanka. Although the original PAQ-C is one of the best assessment tools for this purpose [23], language barriers may affect its accuracy when administered among non-English speaking children [30]. Moreover, differences in cultural and social aspects may also alter the results. However, the PAQ-C has not been translated into the Sinhala language and no existing studies have examined the reliability and validity of the Sinhala version of such a questionnaire. Therefore, the purpose of this study was to culturally adapt the PAQ-C into the Sinhala language and to examine the validity and reliability of the Sinhala version among Sri Lankan children aged 8–14 years.

## Materials and methods

This cross-sectional study was conducted between 2^nd^ May 2023 and 31^st^ July 2023 following the Helsinki Declaration. Ethical approval for this study was obtained from the Ethics Review Committee, Faculty of Allied Health Sciences, University of Peradeniya, Peradeniya, Sri Lanka (No: AHS/ERC/2022/132). An information sheet was provided to the parents, and written informed consent was obtained from each. Only the children whose parents consented to their participation in the study completed the questionnaire. All data collection forms were assigned code numbers, and coded data were used throughout the data management process. Phase I involved the translation of the original PAQ-C into Sinhala, while Phase II focused on assessing the psychometric properties of the translated version. Prior to translation, formal permission was obtained from the original developer, Prof. Kent Kowalski [21].

### Phase I-PAQ-C sinhala translation and cross-cultural adaptation

The questionnaire was culturally adapted following internationally endorsed procedures for translation and validation [31].

***Forward Translation:*** Two translations were made from the original English questionnaire. One of the translators is a senior academic with more than 30 years of experience as a physiologist, who is now residing in Australia. He is a native Sinhala speaker. The other translator is a graduate specialising in English, currently working as a teacher at the English Language Teaching Unit of the Faculty of Allied Health Sciences, University of Peradeniya. She is also a native Sinhala speaker and resides in Sri Lanka.

***Synthesis of Translations:*** A third person who was not involved in the study, but who was familiar with the questionnaire, participated in the synthesis process. Both the translated version and the original questionnaire were compared. Both translators commented that some of the sports mentioned in question one are not practised in Sri Lanka (e.g.,: ice-skating), and because of that, no specific Sinhala terms are present for those. Furthermore, it had been hard to translate adverbs of frequency, such as ‘often’ and ‘very often’, because there is a lack of appropriate Sinhala terms to properly communicate the exact, intended idea. To resolve this issue, phrases which give almost similar meanings to the adverbs of frequency in the original questionnaire were used. For some of the questions and answers, the two translators included different terms which give the same meanings. In such cases, words that seem to be most appropriate for the target population were selected. The synthesised version was sent to the translators for back translation.

***Back Translation:*** Back translation was done by two translators who are native Sinhala speakers. One translator is from a physics background and has lived in the United States for five years. The other translator is an English instructor, teaching in the Faculty of Engineering, University of Peradeniya, Sri Lanka.

The two back translations were reviewed with the original questionnaire and back-translated documents were more or less similar to the original, with minor changes in some of the words. However, the meanings were almost similar. In such cases, the words which seemed easier to understand by the target group were selected.

This version was evaluated by an expert panel including 2 physiotherapists, 2 school teachers, and 2 exercise science experts. The original questionnaire contains 22 sports items as options to select in the first question. Among them, ice skating, cross-country skiing, and ice-hockey/ ringette were eliminated in the Sinhala version, since those are not at all performed in Sri Lanka. Rowing/canoeing and street hockey were also eliminated as they are not very common sports among Sri Lankan schoolchildren. In addition, soccer was removed since it is similar to football. Nine new sports items were newly added to the Sinhala version which are commonly played by Sri Lankan school children, namely, karate, netball, cricket, table tennis, elle, boxing, gymnastics, rugby, and tennis. Finally, there were 25 sports items in the first question of the Sinhala version of PAQ-C. A space was provided to mention other sports activities performed by the child, as in the original questionnaire. The final pre-version was prepared after reviewing all deviations. The content validity index (CVI) was calculated to measure content validity. The explanation of CVI can be found under statistical analysis for content validity.

#### Pilot testing.

The final pre-version of the PAQ-C was pilot-tested among 55 school children from a different school within the same educational zone to assess clarity, readability, and comprehensibility.

### Phase II: Evaluating the psychometric properties of PAQ-C Sinhala Version

The PAQ-C questionnaire was regarded as a formative model questionnaire according to COSMIN (COnsensus-based Standards for the selection of health Measurement INstruments) [32]. For formative models, COSMIN recommends focusing on content validity and reliability, as structural validity and internal consistency are not applicable. Accordingly, we evaluated content validity through expert opinion on item relevance, comprehensibility through a pre-test, and reliability through test-retest to assess the stability of responses over time.

#### Participants.

Data collection was conducted in provincial schools in Gangawata Korale, comprising both males and females. Gangawata Korale is an administrative division that includes urban, rural and estate sector areas, and the participating schools enrol pupils drawn from this mix of residential settings; therefore, the sample includes children from urban, rural and estate sectors within the Peradeniya and is representative of mainstream provincial government‑school pupils in similar central‑region settings. Permission was obtained from the Provincial Director of the Department of Education in Kandy District to conduct the data collection in schools. Children whose native language was Sinhala and who studied in grades 4 and 5, between the ages of 9 and 11 years, were included in the study. In Sri Lanka, Grade 6 marks the transition to secondary school; we therefore limited recruitment to Grades 4 and 5 to focus on primary school children with comparable routines, developmental stages, and comprehension levels suitable for self-administered questionnaires like the PAQ‑C. Children who were involved in regular physical activity were included. Children with acute illnesses, chronic medical condition or disability that prevented them from participation in regular physical activities were excluded. Children whose parents did not grant consent for the participation of their child in the study were also not included.

As a rule of thumb, 10 participants are described as essential for each item in the questionnaire [33]. However, there are suggestions independent of the number of items; it is proposed to have 300 respondents after pre-testing [34]. The sample size was determined as 300 for this study in order to have a good sample for validation of the tool [35]. Two schools were selected randomly as clusters, and participants were selected by convenient sampling within the schools. A total of 330 students were invited to participate in the study, of whom 318 provided informed consent. Seventeen students were excluded in total this including those who, based on their responses to Question 10, reported that their physical activity levels had been affected by illness during the preceding week, as well as others who met additional exclusion criteria. The final analytical sample comprised 301 students.

#### Procedure.

During the first visit, school authorities and children were given a detailed explanation of the study and questionnaire. Then, consent forms were sent with the children to be read and signed by their parents/ guardians. After three days, the researchers visited the schools again to collect the consent forms. After collecting the consent forms, all children who returned signed parental consent forms were assessed. The periods for data collection were decided by the school authorities in order not to cause interruptions to academic activities.

On the day of data collection, the participants were asked to fill out the Sinhala version of the PAQ-C questionnaire, and they were assessed for body weight and height.

***PAQ-C Measure:*** PAQ-C was used for assessing the level of physical activity. This questionnaire comprises ten questions. The first question includes a checklist of common leisure and sports activities. Two supplemental blank spaces are also provided for participants to enter other activities which are not mentioned in the list. Five points Likert scale (no activity – 1, 7 or more days – 5) is used for scoring this item. The mean score of all activities is calculated and is considered as the composite score of question number 1. The next eight questions are regarding activities conducted at different time periods during the day [e.g.,: at recess, lunchtime, after school, etc.] or the week. The composite value obtained by calculating the mean of the scores of the above-mentioned nine items is the overall PAQ-C score. The tenth item is used for identifying whether a sickness or another event has prevented the child from participating in regular physical activities. This item is not included in the calculation of activity scores.

The PAQ-C was delivered to students after explaining the questionnaire in detail. The researchers were there throughout the completion of the questionnaire by the students to provide any clarifications pertaining to the questionnaire if needed. The researchers visited one of the schools two weeks later and 75 participants were randomly selected from the student log of those who filled out the questionnaire earlier and they were requested to fill the questionnaire again to determine the test-retest reliability of the questionnaire. The random selection was conducted using a computer-generated number list applied to the student log maintained during the initial administration. The retest was carried out in the same school environment, under similar conditions, and with the same level of researcher presence and guidance. The retest sample was drawn exclusively from the original respondents at that school and did not differ demographically or contextually from the main sample.

### Statistical analysis

All the analysis was conducted using SPSS version 25 and all the significant tests were conducted at a 5% significance level and 95% confidence interval.

### Content validity

We assessed content validity by calculating item-level (I-CVI) and scale-level (S-CVI) content validity indexes [36]. The I-CVI was determined by ratings from all expert committee members. The expert committee comprised of two physiotherapists, three school teachers and one psychologist. We used a 4-point ordinal scale to rate each question/item based on its relevance to the fundamental concept. In this scale, 1 meant not relevant, 2 indicated somewhat relevant, 3 meant quite relevant, and 4 indicated highly relevant. To calculate the I-CVI, we divided the number of experts rating an item as 3 or 4 by the total number of experts for each item. We then calculated the average I-CVIs of all items to obtain the S-CVI for each question. An I-CVI > 0.78 was considered excellent, and a S-CVI of ≥ 0.80 was deemed acceptable [37].

### Test-retest reliability

The intraclass correlation coefficient (ICC) one-way random-effects model was used for the analysis of test-retest reliability [38], in which a value > 0.70 was considered acceptable [32].

## Results

### Characteristics of participants

A total of 301 children participated in the study. The majority of children were boys (65.4%). Participants’ characteristics are shown in Table 1.

**Table 1:** Participants’ characteristics.

Characteristic	Male		Female		Total	
	Mean	SD	Mean	SD	Mean	SD
Age (years)	9.80	0.83	9.85	0.84	9.81	±0.83
Weight (kg)	28.70	8.54	27.63	7.30	28.33	±8.14
Height (cm)	134.45	8.41	133.23	8.11	134.03	±8.31

### Physical activity pattern

The average score for the PAQ-C was 2.94 ± 0.71. Table 2 presents the analysis of the PAQ-C questionnaire items by male and female participants and for the total sample. The average PAQ-C score for male participants was 3.04 (SD = 0.75), while female participants had an average score of 2.75 (SD = 0.60).

**Table 2:** Descriptive analysis of the PAQ-C questionnaire.

Question	Male		Female		Total	
	Mean	SD	Mean	SD	Mean	SD
**Spare time activity**	1.87	0.85	1.75	0.85	1.83	0.85
**Physical education**	3.03	1.70	2.88	1.51	2.98	1.63
**Recess**	3.13	1.42	2.41	1.32	2.88	1.43
**Lunch**	3.13	1.27	2.52	1.36	2.92	1.33
**After School**	3.09	1.50	2.95	1.35	3.04	1.45
**Evening**	3.47	2.50	3.41	1.26	3.45	2.15
**Weekends**	3.54	1.29	3.39	1.19	3.49	1.26
**Describes you best**	3.19	1.36	2.86	1.24	3.08	1.33
**Activity frequency**	2.88	0.79	2.56	0.64	2.77	0.76

### Validity of the PAQ-C

#### Content validity.

The I‑CVI and S‑CVI were both 1.00, indicating unanimous expert agreement and good content validity in terms of relevance.

### Reliability of the PAQ-C

The test’s reliability was assessed using the ICC. The overall ICC value for the questionnaire was 0.83, with ICC; one-way random-effects mode showing adequate test-retest reliability. The average ICC for each item of the questionnaire is presented in Table 3

**Table 3:** ICC for each item of the questionnaire.

Item number	ICC
1	0.80
2	0.89
3	0.80
4	0.83
5	0.70
6	0.52
7	0.52
8	0.88
9	0.75

## Discussion

The PAQ-C questionnaire was culturally adapted for the Sinhalese population using a sample of 301 schoolchildren. This study provided the first valid measurement tool to examine the physical activity patterns of Sinhala-speaking children in Sri Lanka. The examination of the psychometric properties of the Sinhala version of the PAQ-C demonstrated good content validity in terms of relevance based on expert agreement and comprehensibility based on pretest results.

The translation process adhered to the standardised guidelines proposed by Beaton and Bombardier [31]. The original structure of the questionnaire was retained, with the exception of item one. In this item, activities that were not commonly practised by Sri Lankan children were removed and replaced with culturally relevant and frequently performed alternatives. This approach aligns with the adaptations reported in the Japanese [14], Turkish [27], and Hungarian [17] versions of the PAQ-C, and is consistent with Beaton and Bombardier’s protocol Beaton, Bombardier (32), which emphasises the importance of achieving both semantic and cultural equivalence during cross-cultural translation.

Regarding content validity, the COSMIN guideline highlights three domains: relevance, comprehensibility, and comprehensiveness. In our study, relevance was assessed by calculating the item-level and overall Content Validity Index (CVI). A similar method was used in the cross-cultural validation of the PQC into the Turkish language [27].

We assessed comprehensibility in terms of readability and understandability using a pilot test of 50 students. Similar procedures were used in cross‑cultural adaptations of the PAQ‑C, with a smaller pilot for the Japanese [14], Hong Kong Chinese version [24] and pilot studies for the Brazilian, Arabic [20], Turkish [27] and Spanish [26] versions, while the UK [39] adaptation used a focus group to evaluate clarity and readability. Our retest reliability analysis showed high consistency of the scale over time, with an ICC of 0.83. Similar research on Spanish children revealed an even higher ICC of 0.96 [26], while studies involving Chinese children in Hong Kong reported an ICC of 0.82 [24], and Japanese children showed 0.83 [14]. Additionally recent systematic review has identified the PAQ-C as demonstrating sufficient reliability and the strongest reliability evidence among physical activity questionnaires for children [23]. These results confirm that the scale consistently performs well over time within the target group.

All participants presented a summarised PAQ-C score, which is lower than that in the studies among different racial groups of children, i.e., 3.4 ± 0.68 for British samples [39], 3.36 ± 0.80 for European American children, and 3.37 ± 0.69 for African American children [40], but it was higher than the PAQ-C scores of Japanese children, 2.76 ± 0.69 [14]. This difference may be due to cultural variations in the country. It also indicates that the physical activity level of Sri Lankan children needs improvement. The spare time activity checklist (question 1) of the Sinhala version of PAQ-C has shown the lowest score. Similar observations were made among the British [39] and Turkish schoolchildren [27]. The lower mean for question one is interpreted as a consequence of the large number of activities listed on the checklist. Many children did not perform most of these activities, leading to lower scores [27].Additionally, prior studies in Italian and Spanish cohorts have proposed a PAQ-C score threshold of 2.75 to classify children as active or non-active [41, 42]. Although our study did not apply such thresholds, the validated Sinhala PAQ-C could support future national-level comparisons using these benchmarks.

### Limitation

The study was conducted with 4th and 5th-grade students between the ages of 9 and 11. To apply the study’s findings more broadly, further research should be carried out with school children between the ages of 12 and 14..To enhance the instrument’s accuracy, concurrent validity should be evaluated against an accelerometer; until this is confirmed, researchers should use the questionnaire cautiously or in conjunction with an accelerometer.

Future research may consider employing the Sinhala PAQ-C within longitudinal study designs to monitor physical activity trends over extended periods, evaluate the effectiveness of targeted interventions, and facilitate comparative analyses across geographic regions and demographic subgroups. Furthermore, the integration of internationally recognised thresholds could enhance the potential for cross-cultural comparisons and contribute meaningfully to national physical activity surveillance initiatives.

## Conclusion

This study was the first to thoroughly examine the validity and reliability of the PAQ-C among Sinhala-speaking children. The Sinhala version of the PAQ-C showed an acceptable level of content validity and adequate test-retest reliability. This cost-effective tool will assist researchers and clinicians in assessing physical activity levels among Sinhala-speaking children in Sri Lanka and abroad, enabling them to understand PA levels and take appropriate measures. Its ease of administration and cultural relevance further support its practical use in school-based assessments and community-level health initiatives, enabling broader monitoring of physical activity patterns among Sri Lankan children.

## Supporting information

S1 File PAQC-English Version. The English version of the PAQ-C used in this study.
(PDF)

S2 File PAQ-C-PLOSONE. Dataset used for statistical analysis.
(XLSX)

S3 File Sinhala Questionnaire-final. The Sinhala version of the PAQ-C questionnaire.
(DOCX)
