# Supplementary material for: Reliability and validity of the Sinhala version of the physical activity questionnaire for older children (PAQ-C)
Source: PLoS One. 2026 Mar 23;21(3):e0343709. doi: 10.1371/journal.pone.0343709 (PMC13008094; doi:10.1371/journal.pone.0343709)
Supplement: S1 File — The English version of the PAQ-C used in this study. (PDF) [file pone.0343709.s001.pdf]

Physical Activity Questionnaire (Elementary School)

Name:_________________________ Age:___________

Sex: M_______ F_______ Grade:__________

Teacher:_______________________

We are trying to find out about your level of physical activity from ***the last 7 days*** (in the last week). This includes sports or dance that make you sweat or make your legs feel tired, or games that make you breathe hard, like tag, skipping, running, climbing, and others.

Remember:

1. There are no right and wrong answers — this is not a test.
2. Please answer all the questions as honestly and accurately as you can — this is very important.

---

1. Physical activity in your spare time: Have you done any of the following activities in the past 7 days (last week)? If yes, how many times? (Mark only one circle per row.)

	No	1-2	3-4	5-6	7 times or more
Skipping	❍	❍	❍	❍	❍
Rowing/canoeing	❍	❍	❍	❍	❍
In-line skating	❍	❍	❍	❍	❍
Tag	❍	❍	❍	❍	❍
Walking for exercise	❍	❍	❍	❍	❍
Bicycling	❍	❍	❍	❍	❍
Jogging or running	❍	❍	❍	❍	❍
Aerobics	❍	❍	❍	❍	❍
Swimming	❍	❍	❍	❍	❍
Baseball, softball	❍	❍	❍	❍	❍
Dance	❍	❍	❍	❍	❍
Football	❍	❍	❍	❍	❍
Badminton	❍	❍	❍	❍	❍
Skateboarding	❍	❍	❍	❍	❍
Soccer	❍	❍	❍	❍	❍
Street hockey	❍	❍	❍	❍	❍
Volleyball	❍	❍	❍	❍	❍
Floor hockey	❍	❍	❍	❍	❍
Basketball	❍	❍	❍	❍	❍
Ice skating	❍	❍	❍	❍	❍
Cross-country skiing	❍	❍	❍	❍	❍
Ice hockey/ringette	❍	❍	❍	❍	❍
Other:					
__________________________	❍	❍	❍	❍	❍
__________________________	❍	❍	❍	❍	❍

2. In the last 7 days, during your physical education (PE) classes, how often were you very active (playing hard, running, jumping, throwing)? (Check one only.)

I don't do PE ....❍
Hardly ever ....❍
Sometimes ....❍
Quite often ....❍
Always ....❍

3. In the last 7 days, what did you do most of the time *at recess*? (Check one only.)

Sat down (talking, reading, doing schoolwork)……❍
Stood around or walked around ....❍
Ran or played a little bit ....❍
Ran around and played quite a bit ....❍
Ran and played hard most of the time ....❍

4. In the last 7 days, what did you normally do *at lunch* (besides eating lunch)? (Check one only.)

Sat down (talking, reading, doing schoolwork)……..❍
Stood around or walked around ....❍
Ran or played a little bit ....❍
Ran around and played quite a bit ....❍
Ran and played hard most of the time ....❍

5. In the last 7 days, on how many days *right after school*, did you do sports, dance, or play games in which you were very active? (Check one only.)

None ....❍
1 time last week ....❍
2 or 3 times last week ....❍
4 times last week ....❍
5 times last week ....❍

6. In the last 7 days, on how many *evenings* did you do sports, dance, or play games in which you were very active? (Check one only.)

None ....❍
1 time last week ....❍
2 or 3 times last week ....❍
4 or 5 last week ....❍
6 or 7 times last week ....❍

7. *On the last weekend*, how many times did you do sports, dance, or play games in which you were very active? (Check one only.)

None .... ❍
1 time .... ❍
2 — 3 times .... ❍
4 — 5 times .... ❍
6 or more times .... ❍

8. Which *one* of the following describes you best for the last 7 days? Read *all five* statements before deciding on the *one* answer that describes you.

A. All or most of my free time was spent doing things that involve little physical effort .... ❍

B. I sometimes (1 — 2 times last week) did physical things in my free time (e.g. played sports, went running, swimming, bike riding, did aerobics) .... ❍

C. I often (3 — 4 times last week) did physical things in my free time .... ❍

D. I quite often (5 — 6 times last week) did physical things in my free time .... ❍

E. I very often (7 or more times last week) did physical things in my free time .... ❍

9. Mark how often you did physical activity (like playing sports, games, doing dance, or any other physical activity) for each day last week.

	None	Little bit	Medium	Often	Very often
Monday	❍	❍	❍	❍	❍
Tuesday	❍	❍	❍	❍	❍
Wednesday	❍	❍	❍	❍	❍
Thursday	❍	❍	❍	❍	❍
Friday	❍	❍	❍	❍	❍
Saturday	❍	❍	❍	❍	❍
Sunday	❍	❍	❍	❍	❍

10. Were you sick last week, or did anything prevent you from doing your normal physical activities? (Check one.)

Yes .... ❍
No .... ❍

If Yes, what prevented you? ___________________________________
