# Supplementary material for: Reliability and validity of the Sinhala version of the physical activity questionnaire for older children (PAQ-C)
Source: PLoS One. 2026 Mar 23;21(3):e0343709. doi: 10.1371/journal.pone.0343709 (PMC13008094; doi:10.1371/journal.pone.0343709)
Supplement: S3 File — The Sinhala version of the PAQ-C questionnaire. (DOCX) [file pone.0343709.s003.docx]

**ප්‍රාථමික විද්‍යාල සිසුන් සඳහා ශාරීරික ක්‍රියාකාරකම් පිලිබඳ ප්‍රශ්නාවලිය**

නම :

වයස :

ස්ත්‍රී/පුරුෂ භාවය :

ගුරුතුමා /ගුරුතුමිය :

අප උත්සාහ කරන්නේ පසුගිය දින හත (පසුගිය සතිය ඇතුලත) ඇතුලත ඔබගේ ශාරීරික ක්‍රියාකාරකම් මට්ටම දැන ගැනීමටයි.

ක්‍රීඩා හෝ නැටුම් මෙම ක්‍රියාකාරකම් වලට ඇතුලත් වේ.

මේ සඳහා ඔබට දහදිය දැමීමට හෝ පාද වෙහෙසට පත්වීමට හේතුවන ක්‍රීඩා සහ නර්තනය හෝ ඔබගේ හුස්ම ගැනීම වේගවත් කරවන , ලණු පැනීම ,දිවීම, කදු හෝ ගස් නැගීම හෝ වෙනත් සෙල්ලම් අන්තර් ගත වේ .

වැදගත්

1.මෙහි වැරදි හෝ නිවැරදි පිළිතුරු නොමැත.මෙය පරීක්ෂණයක් නොවේ.

2.කරුණාකර ඔබට හැකිතාක් අවංක හා නිවැරදි ලෙස සියලු ප්‍රශ්න වලට පිළිතුරු ලබා දෙන්න.

1. ඔබගේ විවේක කාලයේදී සිදුකල ශාරීරික ක්‍රියාකාරකම් : ඔබ පසුගිය දින 7 (පසුගිය සතිය) තුල පහත සදහන් ක්‍රියාකාරකම් වලින් කුමන හෝ ක්‍රියාකාරකමක යෙදුනේද ? යෙදුනේනම් කී වරක්ද? (එක් පේළියක එක රවුමක් පමණක් ලකුණු කරන්න.)

නැත 1-2 3-4 5-6 7 හෝ වැඩි

ලණු පැනීම (ස්කිපින්ග්)

කරාටේ

දැල්පන්දු

ක්‍රිකට්

මේසපන්දු එල්ලේ

බොක්සින්

ජිම්නාස්ටික්

රග්බි

ටෙනිස්

රෝද සපත්තු පැදීම

අල්ලන සෙල්ලම

ව්‍යායාම සදහා ඇවිදීම

බයිසිකල් පැදීම

සෙමින් හෝ වේගයෙන් දිවීම එරෝබික්ස් ව්‍යායාම පිහිනීම

බේස් බෝල් හෝ සැහැල්ලු පන්දු ක්‍රීඩාව නර්තන

පාපන්දු

බැඩ්මින්ටන්

ස්කේටින්ග් පුවරුව පැදීම

වොලිබෝල්

ගෘහස්ථ හොකී

පැසිපන්දු

වෙනත් ශාරීරික ක්‍රියාකාරකම්

1. පසුගිය දින හත තුල, ඔබගේ ශාරීරික අධ්‍යාපන පන්ති වලදී කොපමණ වතාවක්, ඔබ ඉතා ක්‍රියාකාරී වී සිටියේද (වෙහෙස වී ක්‍රියාකිරීම, දිවීම ,උඩ පැනීම ,විසි කිරීම)

එකක් පමණක් ලකුණු කරන්න

- මම ශාරීරික අධ්‍යාපනයේ නොයෙදුනෙමි.
- කලාතුරකින්
- සමහරවිට
- බොහෝවිට
- සැමවිට

1. පසුගිය දිනහත තුල පාසල් විවේක කාලයේදී ඔබ බොහෝවිට කළේ කුමක්ද?

එකක් පමණක් ලකුණු කරන්න

- වාඩිවීසිටීම (කතාකරමින් ,කියවමින්,පාසල් වැඩකටයුතු කරමින්)
- සිටගෙන සිටීම හෝ ඇවිදිමින් සිටීම
- මදක් දිවීම හෝ සෙල්ලම් කිරීම
- සෑහෙන ප්‍රමාණයක් දිවීම සහ සෙල්ලම් කිරීම
- බොහෝ වෙලාවක් උපරිම ප්‍රමාණයෙන් දිවීම සහ සෙල්ලම් කිරීම

1. පසුගිය දින හත තුලදී දිවා ආහාර කාලයේ දී (ආහාර ගැනීමට අමතරව) ඔබ සමාන්‍යයෙන් සිදුකළේ කුමක්ද?

(එකක් පමණක් ලකුණු කරන්න )

- වාඩිවීසිටීම (කතාකරමින් ,කියවමින් , පාසල් වැඩ කටයුතු කරමින්)
- සිටගෙන සිටීම හෝ ඇවිදිමින් සිටීම
- මදක් දිවීම හෝ සෙල්ලම් කිරීම
- සෑහෙන ප්‍රමාණයක් දිවීම සහ සෙල්ලම් කිරීම
- බොහෝ වෙලාවක් උපරිම ප්‍රමාණයෙන් දිවීම සහ සෙල්ලම් කිරීම

1. පසුගිය දින හත තුල කොපමණ දිනක් ඔබ පාසැල අවසන්වූ වහාම ඉතා ක්‍රියාශීලීව ක්‍රීඩා, නැටුම් හෝ සෙල්ලම් කළේද ?

- එක් දිනක් හෝ නොමැත
- පසුගිය සතියේ එක් වරක්
- පසුගිය සතියේ දෙවරක් හෝ තුන්වරක්
- පසුගිය සතියේ සිවු වරක්
- පසුගිය සතියේ පස් වරක්

1. පසුගිය දින හත තුල කොපමණ දිනක් ඔබ සවස් කාලයේදී ඉතා ක්‍රියාශීලීව ක්‍රීඩා, නැටුම් හෝ සෙල්ලම් කළේද ? (එකක් පමණක් ලකුණු කරන්න )

- එක් දිනක් හෝ නොමැත
- පසුගිය සතියේ එක් වරක්
- පසුගිය සතියේ දෙවරක් හෝ තුන්වරක්
- පසුගිය සතියේ සිවු වරක් හෝ පස්වරක්
- පසුගිය සතියේ හයවරක් හෝ හත්වරක්

1. පසුගිය සති අන්තයේ කොපමණ වරක් ඉතා ක්‍රියාශීලීව, ක්‍රීඩා, නැටුම් හෝ සෙල්ලම් කළේද ? (එකක් පමණක් ලකුණු කරන්න )

- එක් වතාවක් හෝ නොමැත
- එක්වරක්
- 2-3 වරක්
- 4-5 වරක්
- 6 වරක් හෝ වැඩි වාර ගණනක්

1. පහත සදහන් කුමන වාක්‍යයක්, පසුගිය දින හත තුල ඔබව හොදින්ම විස්තර කරයිද ? ඔබේ පිළිතුර සටහන් කිරීමට ප්‍රථම පහත සඳහන් වාක්‍ය පහම කියවන්න.

අ : මාගේ මුළු නිදහස් කාලයම හෝ නිදහස් කාලයෙන් බොහෝ වෙලාවක් ගත කලේ අඩුම ශාරීරික ක්‍රියාකාරකම් කරමින්ය

ආ : මගේ නිදහස් කාලයේදී සමහරවිට (පසුගිය සතියේ 1-2 වරක්) ශාරීරික ක්‍රියාකාරකම්හී නිරත වුයෙමි ( උදා: ක්‍රීඩාවන්හි නිරත වීම , දිවීම, පිහිනීම, බයිසිකල් පැදීම ,එරෝබික් )

ඇ : මම මගේ නිදහස් කාලයේදී නිතරම (පසුගිය සතියේ 3-4 වරක් ) ශාරීරික ක්‍රියාකාරකම්හි නිරත වුයෙමි

ඈ : මම මගේ නිදහස් කාලයේදී සුලබව (පසුගිය සතියේ 5-6 වරක්) ශාරීරික ක්‍රියාකාරකම්හි නිරත වුයෙමි

ඉ : මම මගේ නිදහස් කාලයේදී ඉතාමත් සුලබව (පසුගිය සතියේ 7වරක් හෝ වැඩියෙන්) ක්‍රියාකාරකම්හි නිරත වුයෙමි

1. ඔබ පසුගිය සතිය තුල දිනකට ශාරීරික ක්‍රියාකාරකම් වල (ක්‍රීඩා කිරීම ,සෙල්ලම් කිරීම,නැටුම් හෝ වෙනත් ශාරීරික ක්‍රියාකාරකම්) නිරත වූ වාර ගණන පහත සටහනේ ලකුණු කරන්න

|  | එක වතාවක් හෝ නොමැත | ඉතාමත් සුළුවෙන් | මධ්‍යම ප්‍රමාණයෙන් | නිතරම | ඉතාමත් සුලබව |
| --- | --- | --- | --- | --- | --- |
| සඳුදා |  |  |  |  |  |
| අඟහරුවාදා |  |  |  |  |  |
| බදාදා |  |  |  |  |  |
| බ්‍රහස්පතින්දා |  |  |  |  |  |
| සිකුරාදා |  |  |  |  |  |
| සෙනසුරාදා |  |  |  |  |  |
| ඉරිදා |  |  |  |  |  |

1. ඔබ පසුගිය සතියේ අසනීප තත්වකින් පෙළුනාද , එසේත් නොමැති නම් කුමක් හෝ හේතුවක් නිසා ඔබගේ සාමාන්‍ය ශාරීරික ක්‍රියාකාරකම් වැළකුනාද

(එකක් ලකුණු කරන්න)

ඔව්

නැත

ඔව් නම් එසේ වීමට හේතුව කුමක්ද? ………………………………………………………..
